# Successful Incorporation of High Quality Left Ventricular Global Longitudinal Strain Into the Workflow of a Regional Hospital Echocardiography Laboratory

**DOI:** 10.1111/echo.70452

**Published:** 2026-04-13

**Authors:** Nour Beydoun, Madeline Jankowski, Asra Azizuddin, Kristina Degesys, Raja Mutharasan, Nauman Mushtaq, Brian Fey, Diane Smith, Zhiying Meng, Abigail S. Baldridge, Nausheen Akhter, Vera H. Rigolin

**Affiliations:** ^1^ Northwestern University Feinberg School of Medicine Chicago Illinois USA; ^2^ Northwestern Medicine Chicago Illinois USA

**Keywords:** audits with feedback, echo quality improvement, left ventricular global longitudinal strain, PDSA cycle

## Abstract

**Background:**

As hospitals merge with large healthcare systems, maintaining quality across all echocardiography laboratories has increased in complexity. Since left ventricular global longitudinal strain (LV‐GLS) is now available for routine use, some small echo labs may not be performing this technique routinely. We demonstrate the feasibility of an educational intervention accompanied by audit and feedback cycles for sonographers and physicians in a regional hospital to incorporate LV‐GLS into their clinical workflow.

**Methods:**

Two physicians and two sonographers with expertise in LV‐GLS performed a baseline audit of randomly selected transthoracic echocardiograms with LV‐GLS performed at a regional hospital. Technical aspects of LV‐GLS acquisition by sonographers and physician LV‐GLS reporting skills were scored. Audit results with feedback were shared with the echo lab members. Three pre‐recorded educational modules were provided for on‐demand viewing. Two subsequent audits and feedback cycles were performed at 2‐ and 5‐months post baseline along with surveys to assess balance measures.

**Results:**

The number of sonographers and physicians evaluating LV‐GLS increased following the educational intervention and each subsequent audit. The overall median score for physicians improved from 42% (audit 1) to 67% (audit 2) to 100% (audit 3), (*p* < 0.01). The overall median score for sonographers showed a trend toward improvement from 79% (audit 1) to 81% (audit 2) to 85% (audit 3), (*p* = 0.11). Surveys revealed that despite LV‐GLS images increasing exam duration, neither sonographers nor physicians felt that LV‐GLS negatively impacted their efficiency, while both felt it added value to their skillset.

**Conclusion:**

Providing educational modules and repeat audits with feedback is a feasible method to incorporate new technology into a regional hospital echo lab and is associated with improved performance. This technique can be used to ensure high‐quality echo standards across a healthcare network as hospital systems continue to expand.

## Graphical abstract

The scores for physicians’ interpretation of different LV‐GLS metrics are shown in this figure for all 3 audits. Scores in blue represent percentage physician scores for each metric in audit 1, scores in yellow represent audit 2, and scores in red represent audit 3. The total scores are the average scores for physicians for all metrics for each audit, and are reported as percentage scores, with the scores seen improving from audit 1 to audit 3

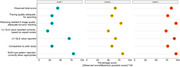

## 1 Introduction

Echocardiography is a routinely used ultrasound imaging modality that aids in the diagnosis and guides treatment of cardiovascular diseases. Ensuring a high level of quality in echocardiography laboratories is vital to ensure optimal patient outcomes. With the evolving field of echocardiography and the constant introduction of new techniques, it is important for echocardiography laboratories to maintain quality standards by obtaining laboratory accreditation, having optimal equipment availability, and providing continuing education for sonographers and physicians [1]. The Intersocietal Accreditation Commission (IAC) guidelines for adult echocardiography accreditation requires facilities to have a quality improvement program and oversight for all imaging procedures [2]. This includes a technical quality review for the sonographers and interpretive quality review for the physicians [2]. As hospitals merge with larger healthcare centers, maintaining quality and standardization across echocardiography labs within a healthcare system has increased in complexity. Optimal strategies for maintaining quality and advancing standardization, particularly related to uptake of new techniques, remain undefined.

Left ventricular global longitudinal strain (LV‐GLS) is a tool developed to detect subclinical myocardial dysfunction, and many commercially available vendors have developed speckle tracking software for strain analysis [3]. LV‐GLS is now available for routine clinical use in most echo systems. However, echo labs in some hospitals may not be regularly performing this technique. We demonstrate the feasibility of an intervention consisting of educational modules along with audit and feedback cycles to assist the echo lab at a recently integrated Northwestern Medicine regional hospital to incorporate evaluation of LV‐GLS into routine clinical practice. Although this project focuses on strain imaging, the implementation strategy described is broadly applicable to emerging techniques in other cardiovascular imaging laboratories.

The goal of this project was to test whether the PDSA (Plan‐Do‐Study‐Act) method is feasible and effective in teaching new technology in an echocardiography laboratory. The PDSA cycle is a quality improvement approach that provides a structured iterative development of change which has demonstrated improvement in patient outcomes and care [4]. The PDSA method has not been commonly used as a process improvement framework to help introduce new technology in an echocardiography laboratory. We chose this method for our study given that the intervention can be planned and tested on a small scale, and allows for continuous data collection, which facilitates rapid feedback leading to adaptations.

In this study we report the use of the PDSA methodology consisting of the use of educational modules accompanied by audit and feedback cycles to assist an echocardiography laboratory in appropriately incorporating LV‐GLS into their clinical workflows.

## 2 Methods

### 2.1 Study Site

The Northwestern Medicine hospital system is composed of 11 hospitals including 1 academic medical center and 10 regional community and rehabilitation hospitals across the greater Chicago metropolitan area. This study was done at the echocardiography laboratory at Northwestern Medicine Palos Hospital, which is a regional affiliate hospital in the southwest Chicago suburbs. All echocardiograms were performed using the Phillips EpiQ system with integrated speckle tracking software (Philips EpIQ, Andover, MA), which was used uniformly across all examinations. LV‐GLS was performed on‐cart for all examinations. Offline calculation of LV‐GLS was also possible using specialized software integrated into the echo workstation (Tomtec, Munich, Germany). IRB approval was waived by the Northwestern University IRB since this was a quality improvement project.

### 2.2 Educational Intervention

Three pre‐recorded educational modules (“strain basics,” “how to acquire and interpret strain images,” and “illustrative cases”) were provided on demand after the first audit was completed and were viewed at least once by all Palos sonographers and physicians. Verification of video review was based on the honor code. Video content was created based on previous educational materials used to successfully introduce strain imaging in the Northwestern Medicine main academic center. The illustrative cases module was added based on feedback that case‐based learning is a popular and effective method.

### 2.3 Audits

Transthoracic echocardiograms performed at Palos Hospital wherein LV‐GLS was evaluated were randomly selected at three time points from the NM echocardiography database. The baseline, two‐month follow up, and five‐month follow up audits included echocardiograms performed between November 1, 2022 to April 1, 2023; November 1, 2023, to November 15, 2023; and February 15, 2024 to April 2, 2024, respectively. The echocardiograms were randomly selected from a list of all echocardiography studies containing strain within audit period, and were stratified by reading physicians and performing sonographers. From that list, at least two studies were randomly chosen for each physician and each sonographer, assuring equal representation from every NM sonographer and physician working at the Palos echo lab at the time of each audit.

Each audit was performed by the same two physicians and two sonographers with expertise in LV‐GLS. The four experts were not members of the Palos echo lab. At each audit, each selected echocardiogram image and the associated report was carefully reviewed and scored by a pre‐defined rubric which focused on technical aspects of LV‐GLS acquisition by sonographers and physician LV‐GLS reporting skills. A total score for each member was then tabulated (Table 1). For sonographers, the composite score included assessment of non‐foreshortened apical image acquisition, optimization of tracking quality in each apical view, and accurate landmark placement for LV‐GLS measurements, amongst other variables detailed in Table 1. For physicians, the composite score evaluated tracing quality for reporting, appropriate retracing when tracking was suboptimal, accurate reporting of LV‐GLS values, correct inclusion of the bull's‐eye pattern when indicated, and inclusion of a comparison statement to prior studies that appropriately addressed strain findings (further details in Table 1). To note, these scoring elements were not previously piloted or validated; to our knowledge, this is the first study to include these specific variables within a structured QI framework. Review of studies for all three audits were performed collectively by all four experts and scoring made by consensus agreement. Studies were reviewed once during each audit cycle and were not re‐reviewed in subsequent audits.

**TABLE 1:** Scoring system used to audit echocardiograms acquired and interpreted by sonographers and physicians.

Scoring system for sonographers	Points	Scoring system for physician readers	Points
Apical images not foreshortened in each apical view	3	LV GLS value reported	1
Adequate tracking quality in each apical view	6	Tracing quality adequate for reporting	1
Apical images optimized for GLS analysis in each apical view	12	Retracing needed if image quality adequate but poor tracking	1
Landmarks correct for GLS measurements in each apical view	9	LV GLS reported correctly based on expert review	1
>70% segments seen in apical views used for GLS measurements	1	Bulls eye pattern correctly reported when appropriate	1
		Comparison to prior study noted in reports	1
Total score per study:	31	Total score per study:	6

### 2.4 Feedback

Results of each audit were reviewed in an unblinded fashion with the Palos lead sonographer and the medical director who then shared the results with individual sonographers and physicians. An example of audit results is shown in Supplemental Table 1. Audit results and feedback were provided immediately after audit completion to allow time for incorporation of feedback before the next audit. At the end of the study, final results were shared in a blinded fashion to all members of the echo lab.

### 2.5 Survey

**FIGURE 1:**
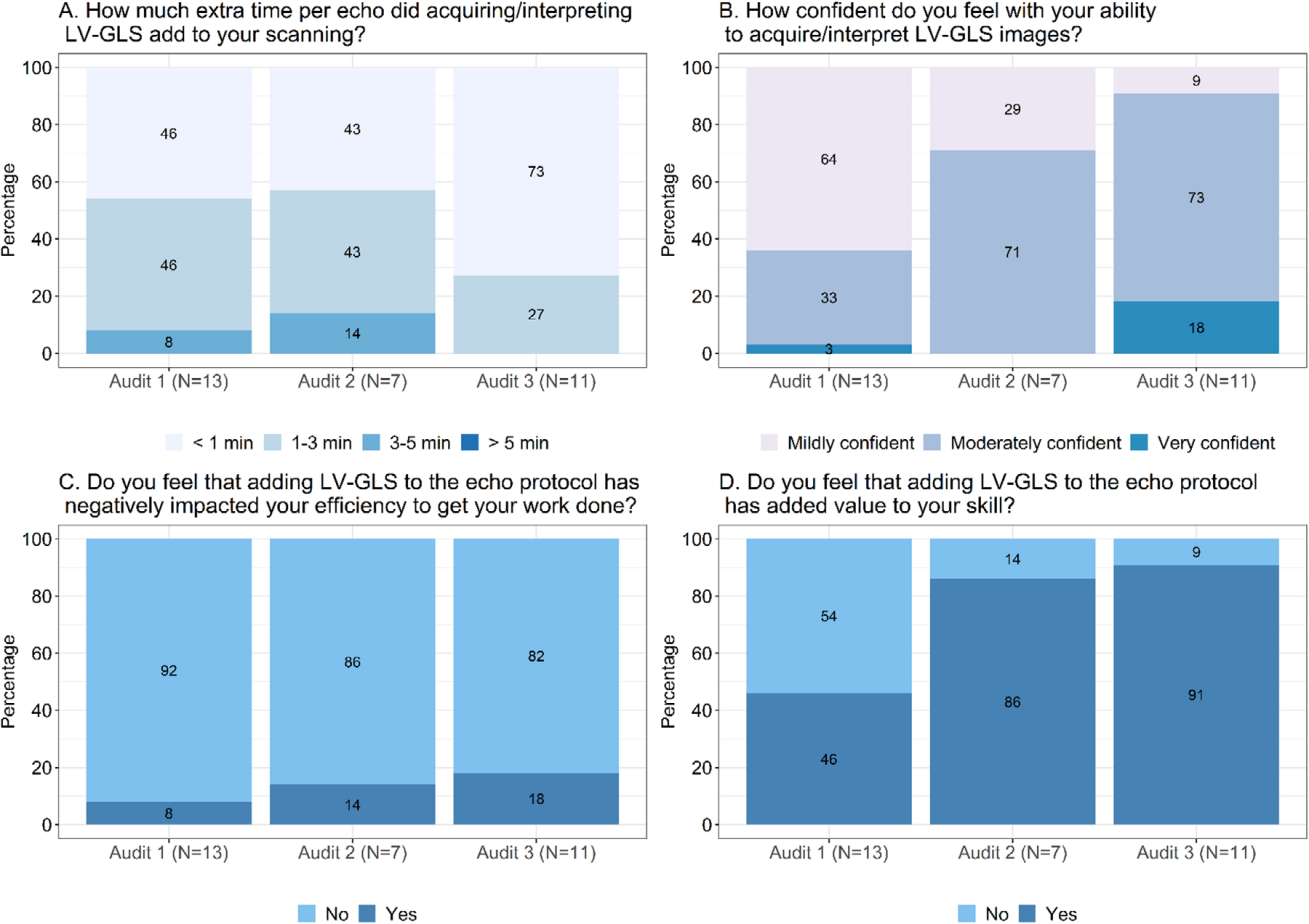
Survey administered after each audit to physicians interpreting left ventricular global longitudinal strain. Results from the surveys administered to physicians interpreting LV‐GLS after each audit are shown in this figure using stacked bar charts with components' proportional contributions. The survey contained four key questions (A, B, C, and D), and physician responses were compared from audits 1 through 3 for each question. The number of physicians participating in the surveys at each audit is indicated.

**FIGURE 2:**
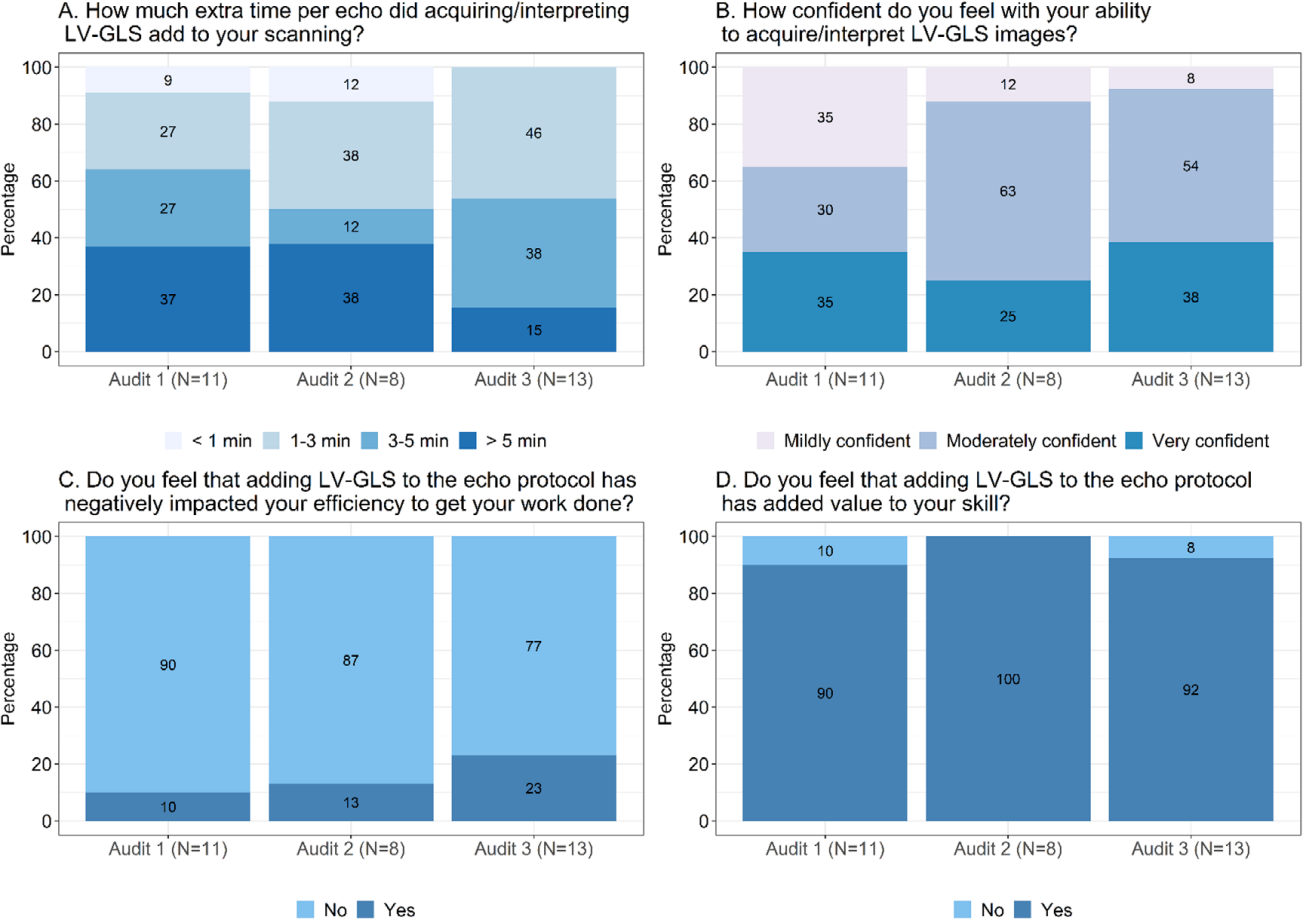
Survey administered after each audit to sonographers acquiring left ventricular global longitudinal strain. Results from the surveys administered to sonographers acquiring LV‐GLS after each audit are shown in this figure using stacked bar charts with components' proportional contributions. The survey contained four key questions (A, B, C, and D), and sonographers’ responses were compared from audits 1 through 3 for each question. The number of sonographers participating in the surveys at each audit is indicated.

A survey was also administered at baseline after the educational intervention and after each audit to assess balance measures. Content of the surveys can be seen in Figures 1 and 2.

### 2.6 Statistical Methods

Physician score elements were recorded as binary correct or incorrect and were summarized using frequency and percentages and were compared between audits using Chi‐squared tests. Sonographer score elements were computed against a response bank ranging from 0 to 4 points and indexed to a possible 100% accuracy. Median and interquartile range were used for continuous sonographer score elements and compared between groups using Kruskal–Wallis tests. Total score was standardized to a 100‐percentage scale by dividing the observed score by the highest possible score and multiplying the result by 100. We compared median total scores between audits using Kruskal–Wallis tests. Analyses were performed using SAS (Base 9.4, Cary, NC, USA) and R (version 3.5.2, R Core Team, Vienna, Austria) with *p* values <0.05 considered statistically significant.

## 3 Results

At the beginning of the study, there were a limited number of sonographers performing and physicians interpreting LV‐GLS. However, following our educational intervention, the number of sonographers and physicians performing and interpreting LV‐GLS increased. Audit 1 consisted of 10 echocardiograms performed by five sonographers and interpreted by four physicians. Audit 2 consisted of 24 echocardiograms performed by 14 sonographers and interpreted by seven physicians. Audit 3 consisted of 26 echocardiograms performed by 14 sonographers and interpreted by 10 physicians. To note, additional physicians and sonographers were incorporated during subsequent audit cycles (therefore did not benefit from participation in earlier audit cycles), however no participants were removed or excluded throughout the study period.

**FIGURE 3:**
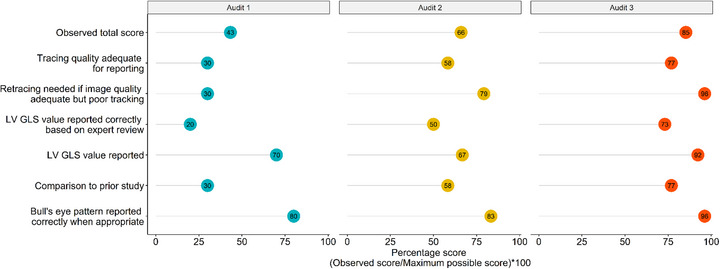
Quality metric scores for physicians’ interpretation of left ventricular global longitudinal strain across audits. The scores for physicians’ interpretation of different LV‐GLS metrics are shown in this figure for all three audits. Scores in blue represent percentage physician scores for each metric in audit 1, scores in yellow represent audit 2, and scores in red represent audit 3. The total scores are the average scores for physicians for all metrics for each audit, and are reported as percentage scores, with the scores seen improving from audit 1 to audit 3.

**FIGURE 4:**
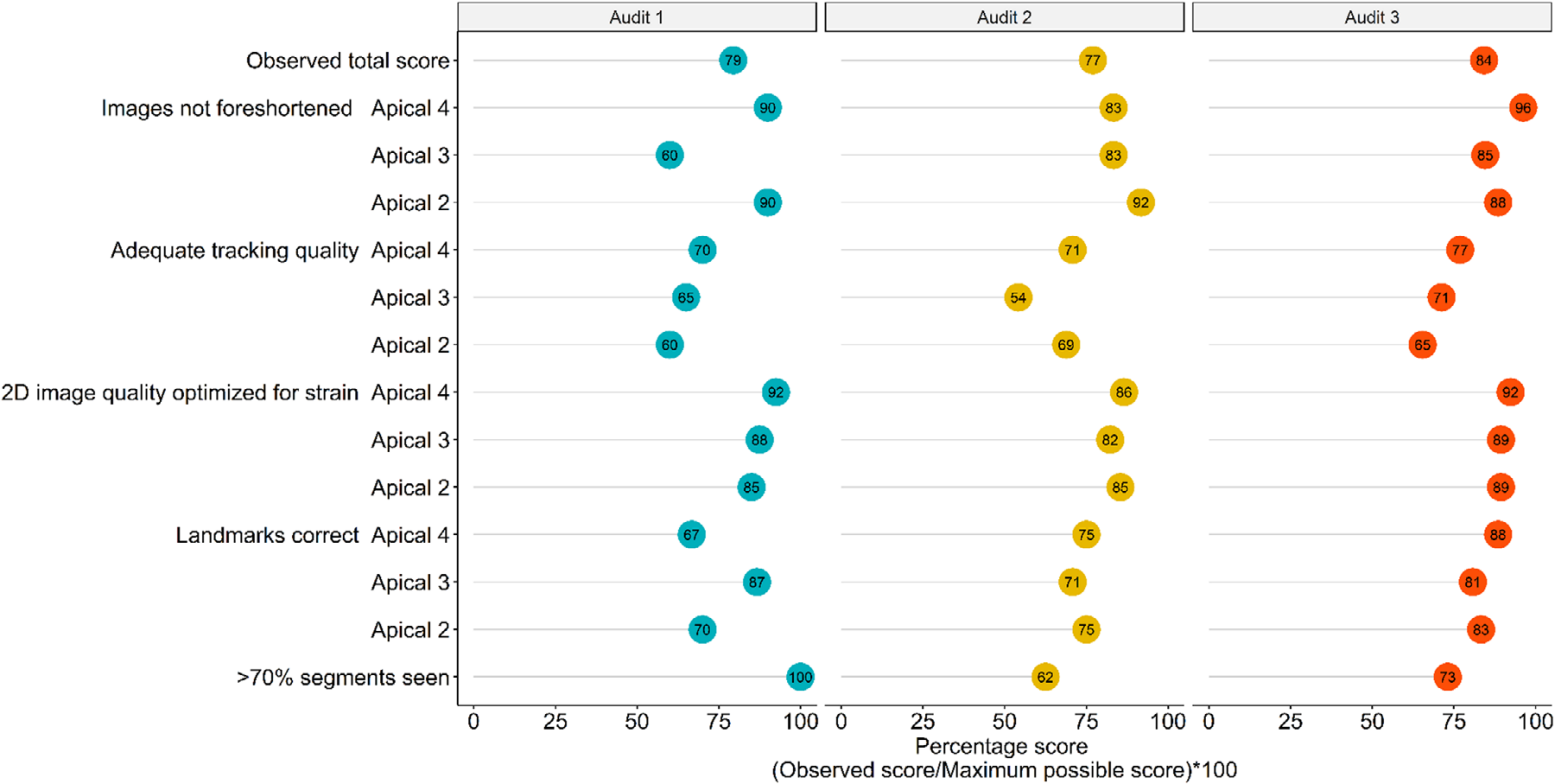
Quality metric scores for sonographers’ acquisition of left ventricular global longitudinal strain across audits. The scores for sonographers’ interpretation of different LV‐GLS metric are shown in this figure for all three audits. Scores in blue represent percentage sonographer scores for each metric in audit 1, scores in yellow represent audit 2, and scores in red represent audit 3. The total scores are the average scores for sonographers for all metrics for each audit, and are reported as average scores, with the scores seen improving from audit 1 to audit 3.

Percentage of correct scores for all three audits for physicians are shown in a graphic format in Figure 3, and data used for this figure is shown in Table S2. The percentage of correct scores for all physician metrics improved from audit 1 to audit 3, with the total score improving from 43% in audit 1 to 85% in audit 3. Percentage of correct scores for all three audits for sonographers are shown in a graphic format in Figure 4, and data used for this figure is shown in Table S3. The percentage scores for most sonographer metrics improved from audit 1 to audit 3, with the total score improving from 79% in audit 1 to 84% in audit 3. Table 2 demonstrates that the overall median score for physicians improved from 42% (audit 1) to 67% (audit 2) to 100% (audit 3), (*p* < 0.01). Table 3 demonstrates that the overall median score for sonographers showed a trend towards improvement from 79% (audit 1) to 81% (audit 2) to 85% (audit 3), (*p* = 0.11).

**TABLE 2:** Quality metric scores for physicians’ interpretation of left ventricular global longitudinal strain across audits.

	Across all time points		By audit			
Correct response for evaluation criterion, no. (%)^a^	*N* (*n* = 60)	Descriptive	1st (*n* = 10)	2nd (*n* = 24)	3rd (*n* = 26)	*p* value^b^
Was the LV GLS value reported?	60	47 (78.3)	7 (70.0)	16 (66.7)	24 (92.3)	0.07
Was the tracing quality adequate for reporting?	60	37 (61.7)	3 (30.0)	14 (58.3)	20 (76.9)	0.031
Was retracing needed?^c^	60	47 (78.3)	3 (30.0)	19 (79.2)	25 (96.2)	<0.01
Was the LV GLS value reported correctly?	60	33 (55.0)	2 (20.0)	12 (50.0)	19 (73.1)	0.013
Was the bull's eye pattern reported correctly (when appropriate)?	60	53 (88.3)	8 (80.0)	20 (83.3)	25 (96.2)	0.25
Was there a comparison to prior study?	60	37 (61.7)	3 (30.0)	14 (58.3)	20 (76.9)	0.031
Total score,^d^ median (IQR)	60	83 (50–100)	42 (33–67)	67 (33–100)	100 (67–100)	<0.01

a Each criterion was scored as correct or incorrect, the proportion of correct scores are reported.

b Chi‐squared test or Kruskal–Wallis tests.

c If image quality was adequate but there was poor tracking or incorrect landmarks.

d Total score was calculated as the number correct out of a possible score of 6 multiplying by 100.

**TABLE 3:** Quality metric scores for sonographers’ acquisition of left ventricular global longitudinal strain across audits.

	Across all time points		By audit			
Score for each evaluation criterion, median (IQR)^a^	*N* (*n* = 60)	Descriptive	1st (*n* = 10)	2nd (*n* = 24)	3rd (*n* = 26)	*p* value^b^
>70% segments seen	60	100 (0–100)	100 (100–100)	100 (0–100)	100 (0–100)	0.08
Apical 2: Images not foreshortened	60	100 (100–100)	100 (100–100)	100 (100–100)	100 (100–100)	0.96
Apical 2: Landmarks correct	60	100 (67–100)	67 (33–100)	67 (67–100)	100 (67–100)	0.31
Apical 2: Adequate tracking quality	60	50 (50–100)	50 (50–100)	75 (50–100)	75 (50–100)	0.83
Apical 2: 2D image quality optimized for strain	60	100 (75–100)	75 (75–100)	75 (75–100)	100 (75–100)	0.34
Apical 3: Images not foreshortened	60	100 (100–100)	100 (0–100)	100 (100–100)	100 (100–100)	0.23
Apical 3: Landmarks correct	60	67 (67–100)	100 (67–100)	67 (50–100)	83 (67–100)	0.19
Apical 3: Adequate tracking Quality	60	50 (50–100)	75 (50–100)	50 (50–50)	100 (50–100)	0.14
Apical 3: 2D image quality optimized for strain	60	100 (75–100)	88 (75–100)	75 (75–100)	100 (75–100)	0.30
Apical 4: Images not foreshortened	60	100 (100–100)	100 (100–100)	100 (100–100)	100 (100–100)	0.33
Apical 4: Landmarks correct	60	100 (67–100)	67 (33–100)	100 (33–100)	100 (67–100)	0.050
Apical 4: Adequate tracking quality	60	100 (50–100)	75 (50–100)	100 (50–100)	100 (50–100)	0.63
Apical 4: 2D image quality optimized for strain	60	100 (75–100)	100 (75–100)	100 (75–100)	100 (100–100)	0.13
Total score^c^	60	81 (74–90)	79 (74–81)	81 (68–87)	85 (77–97)	0.11

a Each evaluation criteria was scored against a response bank ranging from 0 to 4 points. Scores were indexed to a possible 100% accuracy and are reported here as the median score for each item.

b Kruskal–Wallis tests.

c Total score was calculated as the sum of each element against a possible score of 31 and were indexed to a possible 100% accuracy.

Although overall improvement was seen in all audit scores for both sonographers and physicians, there were certain elements where improvement in scores was less robust, thus identifying areas of opportunity for continued improvement. Such elements for physicians included correctly identifying adequate LV‐GLS tracing quality for reporting, reporting LV‐GLS value correctly based on expert review, and adding information on LV‐GLS in the comparison statement to the prior echo (Figure 3). For sonographers, elements identified as opportunities for continued improvement included adequate tracking quality in the apical 2, apical 3, and apical 4 chamber views, and landmarks identified correctly in the apical 3 chamber view (Figure 4).

The surveys revealed that despite LV‐GLS images adding time to the examination, neither sonographers nor physicians felt that LV‐GLS negatively impacted their efficiency, and the majority agreed that it added value to their skillset (Figures 1 and 2). Among physicians, 54% initially reported that interpreting LV‐GLS added more than 1 minute per echocardiogram, however by audit 3 this proportion had decreased to 27%, with the majority (73%) reporting it took less than 1 min (Figure 1). Confidence also improved over time with only 36% of physicians reported feeling moderately to very confident in interpreting LV‐GLS in audit 1, which improved to 91% in audit 3. Similarly by audit 3, 91% of physicians felt that adding LV‐GLS to the echocardiography protocol added value to their skillset (Figure 1). For sonographers, 64% initially reported that acquiring LV‐GLS added more than 3 min per echocardiogram, however this decreased to 53% by audit 3, with only 15% reporting that acquisition took more than 5 min (Figure 2). Confidence also increased significantly, with 65% of sonographers reported being moderately to very confident in acquiring LV‐GLS images, which improved to 92% by audit 3 (Figure 2).

## 4 Discussion

This study demonstrated the feasibility and effectiveness of incorporating LV‐GLS technology using the PDSA method into the workflow of an NM regional hospital echocardiography laboratory that was not previously using this technology. The project aimed not only to incorporate strain technology, but to ensure that studies met quality standards necessary for reliable clinical use. The novel findings of this study are: (1) the significant improvement in physician interpretation of LV‐GLS, (2) the observed trend toward improvement in sonographer acquisition of LV‐GLS, (3) implementation without decline in workflow efficiency, and (4) improved confidence of both physicians and sonographers with perceived value among participants.

The PDSA method is a quality improvement approach that provides a structured iterative development of change. It is based on a four‐stage cyclic learning approach: the “plan” stage where the issue is identified and the initial intervention is designed, the “do” stage where the intervention is tested, the “study” stage where the results are examined to determine the success of the intervention, and the “act” stage where the intervention is refined and the next steps are identified to inform a new cycle [4]. In our study, three PDSA cycles were completed to achieve the study goal (Table 4).

**TABLE 4:** The three cycles of the plan‐do‐act‐study model used in our study.

**PDSA Cycle 1**:
Plan: Incorporation of LV‐GLS proposed to the Palos echo lab
Do: Baseline audit of echo studies containing LV‐GLS
Study: Results of the audit with feedback provided to sonographers and physicians at the Palos echo laboratory
Act: LV‐GLS image acquisition and interpretation adjusted according to audit results with feedback
**PDSA Cycle 2**:
Plan: Continued expansion of LV‐GLS imaging in the Palos echo lab
Do: Educational modules covering key aspects of LV‐GLS image acquisition and interpretation provided to the members of the Palos echo lab followed by Audit 2 of echo studies containing LV‐GLS
Study: Results of the audit with feedback provided to sonographers and physicians at the Palos echo laboratory
Act: LV‐GLS image acquisition and interpretation adjusted according to audit results with feedback
**PDSA Cycle 3**:
Plan: Continued expansion of LV‐GLS imaging in the Palos echo lab
Do: Audit 3 of echo studies containing LV‐GLS
Study: Results of the audit with feedback provided to sonographers and physicians at the Palos echo laboratory
Act: LV GLS image acquisition and interpretation adjusted according to audit results with feedback. Ongoing internal QA program initiated

The PDSA method has been successfully used in different areas of healthcare to implement change, with examples that include improving the assessment of iron deficiency in patients with heart failure [5], reducing catheter‐associated urinary tract infections in the cardiac intensive care unit [6], and improving compliance with hyperlipidemia screening in an outpatient pediatric cardiology clinic [7]. To our knowledge, this is the first study to utilize the PDSA method to introduce novel echocardiography technology into an echocardiography laboratory.

According to the Kaiser Family Foundation, about 1500 hospitals were targeted as part of an acquisition or merger from 2010 through 2019 [8]. The share of community hospitals that are part of a larger health system increased from 53% in 2005 to 68% in 2022 [9]. A similar trend is seen across all healthcare systems in the United States. One key issue has been maintaining quality and standardization across echocardiography laboratories within a healthcare system as it expands and integrates with smaller hospitals. To successfully introduce new technology, continuous education and training are vital, in addition to a quality assurance program and an evaluation process that ensures that echocardiograms are performed and interpreted appropriately [10].

In our study, we achieved successful implementation of high‐quality LV‐GLS by providing educational modules and a series of audits with feedback over time to sonographers and physicians. The educational modules provided fund of knowledge while the audits with feedback allowed for continued process improvement that resulted in improved quality. Audit with feedback is a well‐recognized and effective learning strategy used in quality improvement to implement change [11]. In our study, audit with feedback was done over increasing time intervals, since spaced repetition, a strategy that involves revisiting information at spaced intervals over time, is another recognized effective learning technique that has been successfully used in medical education [12, 13]. We combined both these learning techniques to successfully incorporate LV‐GLS into the echocardiography laboratory at Palos Hospital.

We demonstrated that by using the PDSA technique, there was increased performance and interpretation of LV‐GLS, improvement in acquiring adequate echo images by sonographers and accurate interpretation by physicians, while adding to both sonographers and physicians’ skillsets without significantly impacting their workflow. The echocardiography laboratory at Palos Hospital is now routinely performing LV‐GLS on the appropriate patients.

It is important to note that sonographers demonstrated high baseline performance in image acquisition domains (e.g., obtaining non‐foreshortened images and optimizing image quality for strain), likely reflecting established competencies in these foundational skills. However, strain‐specific components (e.g., adequate tracking quality and correct landmark placement), had comparatively lower baseline scores, allowing identification of targeted opportunities for improvement. In contrast, physician performance reflected assessment of entirely new knowledge domains related to strain interpretations, whereas sonographers encompassed both previously established imaging skills and newly acquired strain‐specific competencies. This difference in baseline skill composition may help explain the differential magnitude of improvement observed between the physician and sonographer groups, with physician scores showing statistically significant improvement, while sonographers scores showing a trend toward improvement.

Although there was marked improvement in several metrics in acquiring or interpreting echocardiography images for optimal LV‐GLS analysis, we identified areas in need of continued improvement. Using this methodology, we can focus education and training in the future on those areas, possibly by using additional PDSA cycles. To continue the improvements achieved, the educational videos remain available on a shared network channel to be reviewed on demand by sonographers and physicians, especially as new members join this echocardiography laboratory.

Our approach emphasizes the importance of continuing education rather than providing a one‐time education session. This quality improvement model may serve as a template to improve quality across echo labs within healthcare systems and can be broadly applicable to the introduction of novel technologies and workflows in other cardiovascular imaging laboratories that encounter similar challenges around training, workflow integration, and quality assurance. Echocardiography laboratories should focus on continuous quality improvement to ensure high quality standards [14]. Given the rapidly evolving field of echocardiography, ongoing sonographer and physician education and development are of utmost importance, as emphasized in the guidelines of the American Society of Echocardiography for cardiac sonographer [15], and the Intersocietal Accreditation Commission guidelines for echocardiography [2]. To maintain quality across hospitals that are acquired by larger healthcare systems, there needs to be robust quality improvement and quality assurance initiatives in place, as well as regular auditing programs.

Our study has several limitations. First, this study was done at a single center, thus limiting generalizability of the results. The sample size was also small. Hands‐on training from Northwestern Medicine main academic center was not provided to the Palos echo lab, however, this provided a real‐world scenario that is typically seen in small hospitals where learning new technology is reliant upon existing staff. This study was performed without a control group, which limits causal inference. Finally, the Palos echo lab personnel were aware of the ongoing audits, therefore may have been subjected to observation bias.

## 5 Conclusion

In conclusion, providing educational modules and repeat audits with feedback using the PDSA model is a feasible and effective method to incorporate new technology in a regional hospital echocardiography laboratory that was not previously using this technology. While the PDSA method has been widely used in quality improvement in healthcare, it is not commonly used in echocardiography. In our study, we demonstrated that this is a feasible method to gain knowledge and reinforce new technology. This method highlights the importance of continuing education rather than providing one‐time teaching. This can be used to ensure high‐quality echo standards across a healthcare network as hospital systems continue to expand. While this project focuses on LV‐GLS, an echocardiographic technique, the structured combination of education and audit‐feedback cycles offers a generalizable framework for implementing new technologies across multiple other cardiovascular imaging laboratories, such as nuclear cardiology, cardiac computed tomography and magnetic resonance imaging.

## Funding

The authors have nothing to report.

## Consent

No written consent has been obtained from the patients as there is no patient identifiable data included in this case report/series.

## Supporting information




**Supporting File 1**: echo70452‐sup‐0001‐SuppMat.docx.
