# Supplementary material for: Successful Incorporation of High Quality Left Ventricular Global Longitudinal Strain Into the Workflow of a Regional Hospital Echocardiography Laboratory
Source: Echocardiography. 2026 Apr 13;43:e70452. doi: 10.1111/echo.70452 (PMC13075460; doi:10.1111/echo.70452)
Supplement: Supplementary file 1 — Supporting File 1: echo70452‐sup‐0001‐SuppMat.docx. [file ECHO-43-e70452-s001.docx]

**Supplemental Table 1: Example of audit feedback given to physicians and sonographers:**

**Feedback given to physicians:**

| Comparison statement is missing. |
| --- |
| For this cardio-oncology patient, the strain should have been re-analyzed due to incorrect placement of annular landmarks. Please include a statement about no prior study for comparison. |
| Would not have reported strain due to poor image quality. |
| Would have retraced strain to better track apical segments. |

**Feedback given to sonographers:**

| Change cardiac cycle in apical 2 chamber so it aligns with other views, tracking not correct in basal anterolateral and anterior segments |
| --- |
| In the apical 3 chamber view, basal tracking is into the mitral valve. Frame rate too high (145) |
| Great job on performing strain on a patient with heart failure even if not ordered. Nice job not including the trabeculation in apical 3 chamber view. |

**Supplemental Table 2. Quality Metric Scores for Physicians’ Interpretation of Left Ventricular Global Longitudinal Strain Across Audits (Percentage Scores)**

| **Variable, %** | **Percentage scores for Audit^1^** | | |
| --- | --- | --- | --- |
|  | **1^st^**  **(n=10)** | **2^nd^**  **(n=24)** | **3^rd^**  **(n=26)** |
| LV GLS value reported | 70 | 66.7 | 92.3 |
| Tracing quality adequate for reporting | 30 | 58.3 | 76.9 |
| Retracing needed if image quality adequate but poor tracking | 30 | 79.2 | 96.2 |
| LV GLS reported correctly based on expert review | 20 | 50 | 73.1 |
| Bulls eye pattern correctly reported when appropriate | 80 | 83.3 | 96.2 |
| Comparison to prior study noted in reports | 30 | 58.3 | 76.9 |
| Total score | 43.3 | 66 | 85.3 |

^1^The score was standardized to a 100-percentage scale by dividing the observed score by the possible maximum score and multiplying the result by 100.

**Supplemental Table 3. Quality Metric Scores for Sonographers’ Interpretation of Left Ventricular Global Longitudinal Strain Across Audits (Percentage Scores)**

| **Variable, %** | **Observed average scores for Audit** | | | |
| --- | --- | --- | --- | --- |
|  | **1^st^**  **(n=10)** | **2^nd^**  **(n=24)** | | **3^rd^**  **(n=26)** |
| >70% segments seen | 100 | | 62.5 | 73.1 |
| Apical 2: Images not foreshortened | 90.0 | | 91.7 | 88.5 |
| Apical 2: Landmarks correct | 70.0 | | 75.0 | 83.3 |
| Apical 2: Adequate tracking Quality | 60.0 | | 68.8 | 65.4 |
| Apical 2: 2D image quality optimized for strain | 85.0 | | 85.4 | 89.4 |
| Apical 3: Images not foreshortened | 60.0 | | 83.3 | 84.6 |
| Apical 3: Landmarks correct | 86.7 | | 70.8 | 80.8 |
| Apical 3: Adequate tracking Quality | 65.0 | | 54.2 | 71.2 |
| Apical 3: 2D image quality optimized for strain | 92.5 | | 86.5 | 92.3 |
| Apical 4: Images not foreshortened | 90.0 | | 83.3 | 96.2 |
| Apical 4: Landmarks correct | 66.7 | | 75.0 | 88.5 |
| Apical 4: Adequate tracking Quality | 70.0 | | 70.8 | 76.9 |
| Apical 4: 2D image quality optimized for strain | 92.5 | | 86.5 | 92.3 |
| Total score | 79.4 | | 77.0 | 84.2 |

^1^The score was standardized to a 100-percentage scale by dividing the observed score by the possible maximum score and multiplying the result by 100.
